# Cook County Medical Society Proceedings for April

**Published:** 1853-05

**Authors:** 


*Cook County Medical Society Proceedings for April.*

[CONTINUED.]

THE subject under discussion being the use of opium in labor, in cases both of convulsions and hemorrhage.

Dr. Clark stated that in cases where the convulsions are hysterical, opium is, so far as he can judge from experience in its use, beneficial.

A case came under his notice, not long since, in which convulsions, with hysterical symptoms, came on during labor, in a patient much debilitated from the bleeding of hemorrhoidal tumors. In this case a full dose of opium procured sleep, and the entire suspension of the convulsions.

Dr. Davis stated that since the last meeting, he had met with a case, in which profuse hemorrhage followed abortion, in a woman at the end of the third month of gestation.

On being called, he found the hemorrhage had already continued to such an extent as to produce coldness of the extremities, and an almost entire absence of pulse at the wrist.

Compression of the æorta arrested the hemorrhage until sufficient time had elapsed to get the effect of a full dose of ergot, which was also administered.

In this case it was found, by experiment, that the hemorrhage did not return when the pressure was removed, even before the effect of the ergot had been obtained.

Dr. Blaney remarked that he had learned from experience that in some cases compression of the aorta is impracticable, in consequence of distension of the abdomen from flatus.

He also gave the history of a case, in which the body of a three months' child had been separated from the head, at the neck, by the midwife in attendance.

On being called, he found the os uteri closed, and the uterus contracted upon the head, which was still within its cavity, with profuse hemorrhage. Matico, by the mouth and in the form of injection, arrested the hemorrhage almost immediately.

In this case, the contracted condition of the os uteri contra-indicated the use of ergot. Gave opium in sufficient quantities to arrest pain and procure sleep.

Three days after, pains returned, and the os uteri became dilated to a sufficient extent to allow of the removal of the head in small pieces, by means of a small pair of forceps. Two weeks after the removal of the head, an apparently fresh placenta was expelled by a return of uterine contractions.

Can call to mind three other cases, in which the placenta was expelled three weeks after abortion.

At the meeting, in May, Dr. Miller gave an account of an interesting case of leucoma, which had recently come under his observation. It was treated mainly by dividing the vessels going to the cornea, first on one side and then on the other. In each case there followed a slight ulceration of the substance of the cornea, after the division of the vessels, which was treated with nit. silver. The sight was perfectly restored.

Dr. Johnson called the attention of the society to the improvement in microscopes by Prof. Riddell, of La. He had recently an opportunity of seeing Prof. R.'s arrangement for binocular vision, and was satisfied that it possessed advantages over the forms heretofore in use.

Dr. Smith related to the society a case of gonorrhea, in which the manner of contracting the disease was a question involving the respectability of a respectable (?) man. H.

